# Clinical evaluation of heart failure patients: what good are biomarkers bringing us?

**DOI:** 10.1007/s12471-014-0520-5

**Published:** 2014-01-31

**Authors:** R. A. de Boer

**Affiliations:** Medical Center Groningen, Department of Cardiology, University of Groningen, PO Box 30.001, 9700 RB Groningen, the Netherlands

The number of biomarker studies in heart failure (HF) is exploding, and while we learn more and more about existing biomarkers and while new biomarkers are constantly being discovered, it is humbling to realise just how little we know and understand of existing biomarkers and their utility.

Currently, biomarkers in heart failure are mainly used to improve diagnostic performance or as a tool in risk stratification and prognostication. Especially for natriuretic peptides (NP), there is strong evidence that using NPs improves diagnosis and strongly predicts prognosis, also when corrected for many covariates and confounders, such as ageing, sex, obesity and renal function [1]. Furthermore, in the last decades, several trials have been conducted to evaluate if NP-guided treatment would be of benefit for the HF patient to improve clinical status (symptoms), but also to improve prognosis. The outcomes of these trials have generated mixed results, but it seems that if one is able to actually drive down NPs below a certain threshold, this may be associated with a better performance and a reduction in hard endpoints [2].

However, when assessing HF patients, regular clinical assessment is common practice, besides diagnosing, prognostication and guiding treatment of patients with HF. We do so because functional status is important for the choice of therapies (e.g. the start of mineralocorticoid receptor antagonists (MRAs), referral for device therapy) [3]. Also, if patients indicate they feel worse, or when physicians categorise patients as having worsened, doctors tend to increase the dose of evidence-based medication (such as ACE inhibitors and beta blockers, provided higher doses are tolerated), or increase the (loop) diuretic dose to relieve symptoms [3]. The value of biomarkers in this more daily routine is less well described and surprisingly few data are available showing how well (or how poorly) biomarkers relate to clinical judgment.

The interesting article by Peeters and colleagues [4] fills in some of these gaps. It is a post-hoc analysis of the Trial of Intensified versus standard Medical therapy in Elderly patients with Congestive Heart Failure (TIME-CHF trial) [5], a trial that randomised HF patients to NP-guided or symptom- guided management. At each visit (baseline, and at 1, 3, 6, 12, and 18 months) investigators performed a clinical assessment, which comprised a functional assessment (functional status, New York Heart Association (NYHA) classification), and physical signs, including oedema, rales, central venous pressure (CVP) and orthopnoea, which were ranked in a 4-rank scale (none, minor, moderate or major). Of note, patients enrolled in TIME-CHF were highly symptomatic, with >75 % of the patients in NYHA class III or higher. Furthermore, at each visit, several biomarkers were measured: NT-proBNP and high sensitivity (hs) troponin, but also more emerging markers such as cystatin-C, hs-CRP, and GDF-15 [6]. The authors aimed to investigate the correlations between these biomarkers and clinical parameters at each time point. Furthermore, in TIME-CHF, NT-proBNP levels were made available to doctors for patients randomised to the NP-guided arm, while they were unavailable for patients randomised to the symptom-guided arm. This made it possible to study whether knowledge of the NT-proBNP levels would affect clinical judgment.

The outcomes showed that overall biomarkers have a poor relation with clinical parameters. NT-proBNP performed best, with the highest correlation coefficients (R) with NYHA class (R between 0.22 and 0.33) and with JVP (R between 0.23 and 0.37). All the other markers had an even more marginal correlation with clinical signs and symptoms.

Most interestingly, the measured correlations between NT-proBNP and NYHA class became stronger as the study progressed, predominantly in the NP-guided arm compared with the symptom-guided arm. On the contrary, this was not observed for physical (and more objective) signs such as JVP and oedema. This strongly suggests that attending physicians were influenced by knowing the NP values, and over time ‘adjusted’ their clinical assessment to match the patients’ NYHA class with the NT-proBNP level that they were given back.

These outcomes underscore how subjective NYHA class is, an observation that all doctors caring for HF patients will acknowledge. This is also supported by the observation by Peeters et al. that NYHA class was strongly confounded by comorbidities and that the relation between NYHA class and NT-proBNP was even weaker in elderly patients – the importance is that in general HF is characterised by multiple comorbidities and is a disease of the elderly. It would have been useful to have been informed on interesting subgroups, for instance patients with very high biomarker levels but with few symptoms, and vice versa, patients with invalidating symptoms whose biomarker values were low. What factors interfere in the dissociation between marker and symptoms and is the prognosis primarily determined by symptoms or by biomarkers? And would other novel biomarkers maybe perform better in this respect [7, 8]?

What does the study by Peeters and colleagues tell us? Should we simply rely on our clinical judgment, since the incremental value of biomarkers for this is futile? To answer this, we should go back to the mere reasons why we measure biomarkers at all. A set of minimum requirements that biomarkers should have in order to satisfy the practising physician has been proposed by Morrow and de Lemos [9]: 1) a biomarker should be measurable at a reasonable cost and the test results should be quickly available to the doctor; 2) a biomarker should provide additional information to the clinical workup and 3) a biomarker should contribute to patient management. It has furthermore been stressed that a biomarker outcome should be interpreted within the entire patient assessment: history, physical examination, and laboratory tests [6, 10]. It seems that at least some of these requirements are met in the study by Peeters et al.

It is my conviction that we misclassify HF patients all the time, and the article by Peeters et al. provides evidence for this. If anything, the biomarker values apparently tell us something different to clinical judgment, given the marginal relations between them. The main question remaining is: what exactly do the biomarkers or biomarker profiles tell us, and is this a tale worth telling or could we do without it? And if a biomarker is disproportionately high for the clinical assessment, what can and should we do?

There are adequately powered well-designed biomarker trials underway (e.g. the Guiding Evidence Based Therapy Using Biomarker Intensified Treatment (GUIDE-IT); ClinicalTrials.gov Identifier: NCT01685840), which will help to determine the utility of biomarkers in HF management. The TIME-CHF investigators show us that we may not judge our HF patients as well as we think we do. Until these trials have been conducted, it seems justified to measure a biomarker (most often: BNP or NT-proBNP) at certain time intervals to supplement our clinical judgment, and maybe act on an unexpected result more often than we anticipate.
